# Relationships of capsule endoscopy Lewis score with clinical disease activity indices, C-reactive protein, and small bowel transit time in pediatric and adult patients with small bowel Crohn's disease

**DOI:** 10.1097/MD.0000000000007780

**Published:** 2017-08-18

**Authors:** Chengcheng He, Jie Zhang, Zhenyu Chen, Xicheng Feng, Zibin Luo, Tianmo Wan, Aimin Li, Side Liu, Yuexin Ren

**Affiliations:** Department of Gastroenterology, Nanfang Hospital, Southern Medical University, Guangzhou, China.

**Keywords:** abbreviated Pediatric Crohn's Disease Activity Index, capsule endoscopy, C-reactive protein, Harvey–Bradshaw Simple Index, Lewis score, Crohn's disease, small bowel transit time

## Abstract

Relationships between the capsule endoscopy Lewis score (LS) and clinical disease activity indices and C-reactive protein (CRP) are controversial in adult patients with Crohn's disease (CD). Also, data on pediatric patients are relatively less. However, correlation between LS and small bowel transit time (SBTT) remains investigational. The aim of the present study was to explore the correlations between LS and clinical disease activity indices, CRP, SBTT in pediatric, and adult patients with small bowel CD.

Retrospective, single-center study on consecutive inpatients with established small bowel CD was conducted. The clinical disease activity index was determined using the abbreviated Pediatric Crohn's Disease Activity Index (aPCDAI) in patients aged <18 years and the Harvey–Bradshaw Simple Index (HBI) in adults. Spearman's rank correlation coefficient was used to assess the correlations of LS with aPCDAI, HBI, CRP, and SBTT, respectively.

150 patients were enrolled (30 children and adolescents). In pediatric patients, correlations between LS and aPCDAI, CRP were moderate (*r*_1_ = 0.413; *r*_2_ = 0.379; *P*_1_ = .023; *P*_2_ = .044). There was no correlation between LS and SBTT (*r* = –0.029; *P* = .88). In adults, weak correlations were found between LS and HBI, SBTT (*r*_1_ = 0.213; *r*_2_ = 0.237; *P*_1_ = .019; *P*_2_ = .009). Correlation between LS and CRP was moderate (*r* = 0.326; *P* < .001). Strong correlations were found between CRP and HBI, aPCDAI (*r*_1_ = 0.522; *r*_2_ = 0.650; *P* < .001). The follow-up patients were all in clinical remission after treatment within 4 months, whereas only a minority reached mucosal healing. HBI, aPCDAI, CRP, and LS in all patients were reduced after treatment, whereas difference in CRP in pediatric patients and difference in LS in adults between baseline and follow-up were not found to be statistically significant. Also, the average SBTT at baseline was not found to be different from that at follow-up in all patients.

The role of capsule endoscopy should be emphasized both in pediatric and adult patients with small bowel CD. Furthermore, the small bowel transit time may not be affected by the grade of small intestinal inflammation.

## 1 Introduction

Crohn's disease (CD) is a chronic immune-mediated inflammatory bowel disease (IBD) with uncertain etiology, which may occur anywhere from the esophagus to the anus in a noncontinuous pattern, but the most common location is the distal ileum. The incidence of CD is found to be the highest in the North America and Europe, whereas there is a sustainable rise in the incidence of Asian CD.^[1]^ Of all the patients with established CD, the lesions of small bowel account for 30% both in pediatric and adult population.^[2,3]^ On account that it is a considerable proportion of CD, the overall assessment of small bowel mucosal inflammation is obligatory. Conventional endoscopy such as colonoscopy and gastroscopy cannot realize the detecting of the entire small bowel mucosal changes. Also, given that small bowel double-balloon endoscopy is time-consuming and may cause certain serious complications, it is not available for effectively small bowel mucosal detecting until the advent of small bowel capsule endoscopy (CE). Since it emerged and was approved the use for evaluating small intestinal lesions in adults by the Food and Drug Administration (FDA) in 2001, small bowel CE has brought lots of benefits so as to strengthen the management of small intestinal inflammation in patients with CD and it has been considered as an effective tool in refinement of mucosal evaluation.^[4–7]^ And in 2004, capsule endoscopy was approved for patients aged between 10 and 18 years old. Both the patency capsule and capsule endoscopy were approved for patients older than 2 years in 2009.^[8]^ CE is a noninvasive and pain-free tool with the capability of detecting the small bowel mucosal changes, which gains a higher diagnostic yield than other radiography modalities such as computed tomography enterography (CTE) or magnetic resonance enterography (MRE) and it is more likely to detect earlier and subtle lesions than other modalities.^[9]^ In the management of patients with CD, in particular, CE plays a crucial role in assessing both disease activity and extent of the entire gastrointestinal tract.

Patients with CD are associated with an increased mortality rate and decreased quality of life in the longtime report.^[10]^ Extra educational and mental stress exert great effect on pediatric patients; therefore, the holistic management of CD involving diagnosis, treatment, and prognosis is particularly important. Also, assessment of disease activity is essential in these aspects. The whole evaluation of CD activity includes clinical, laboratory, and endoscopic assessment. Quantitative measurements are adopted for better analyzing on the grade of inflammation whatever in the clinical or endoscopic management, which yields variable indices. Of all the clinical and endoscopic indices of inflammatory severity, Crohn's Disease Activity Index (CDAI), Harvey–Bradshaw Simple Index (HBI), and Lewis score (LS) are studied and used mostly. LS is an objective and visualized disease activity index, which was developed first in 2008; it involves 3 parameters: villous appearance, ulcer, and stenosis.^[11]^ CDAI is considered as the primary measurement tool in the clinical evaluation of CD, whereas it is complicated and critical for incorporating physical examination and laboratory findings. HBI contains 5 variables and it is convenient and useful in the clinical assessment of CD.^[12]^ Abbreviated Pediatric Crohn's Disease Activity Index (aPCDAI) was developed from the full PCDAI in 2003; it contains 3 history items and 3 physical examination items.^[13]^ Both LS and aPCDAI gain adequate interobserver agreement and formal validation.^[14,15]^ Laboratory parameters such as C-reactive protein (CRP), erythrocyte sedimentation rate (ESR),white blood count, hematocrit, and serum albumin have been all studied from past to now. However, the CRP serum level is wildly used as an inflammatory measurement both in the researches and clinical evaluation.^[16]^

Currently, the goal of CD treatment focus on mucosal healing rather than controlling symptoms exclusively.^[17]^ Since the small bowel CE is considered as an effective tool of detecting small intestinal lesions for more than a decade, it has been widely applied to measure small intestinal inflammation and stratify the degree of mucosal changes in the management of CD. Correlation between endoscopic and clinical disease activity in patients with CD has been studied for many years. Also, a variety of serum activity markers and capsule endoscopic score or clinical score were analyzed in the previous studies, the majority of them explored the correlation between LS and CDAI.^[18–20]^ Controversy exist in their results. Furthermore, the effect of disease grade on the small bowel transit time (SBTT) is conflicting, so correlation between LS and SBTT remains investigational.

The objective of the present study was to observe the small bowel mucosal lesions and stratify the severity of the small intestinal inflammation in pediatric and adult patients with CD by means of the LS. Correlations between LS and HBI, aPCDAI, CRP, and SBTT were analyzed, respectively. The study was also undertaken on the follow-up population.

## 2 Materials and methods

This retrospective, nonrandomized study on consecutive inpatients was undertaken in a single medical center (Nanfang hospital in Guangzhou), between March 2012 to January 2017.

### 2.1 Study population

All the patients were East Asian (Chinese). Inclusion criteria were inpatients with established small bowel CD, those who underwent CE examination and had full information about medical history and specific CRP serum level within 3 days from the time of CE. The diagnosis of CD was based on an overall evaluation, which consists of clinical, biochemical, endoscopic, and histological criteria according to the European consensus for the diagnosis and management of CD.^[21]^ In total, 187 patients fulfilled the inclusion criteria. Patients ineligible for participation were those who had a history of extensive small-bowel resection, known bowel obstruction, ulcerative colitis, indeterminate colitis, gastrointestinal cancer, pregnancy, and any use of nonsteroidal anti-inflammatory drugs (NSAIDs) during 3 months prior to enrollment and incomplete CE examination. At last, 37 patients were excluded because of incomplete CE examinations, so our study enrolled 150 patients.

### 2.2 CE procedure and Lewis score

The CE was performed with MiroCam (IntroMedic Co. Ltd., Seoul, South Korea) and OMOM (Jinshan Science and Technology Co. Ltd., Chongqing, China). Subjects ingested the disposable CE after a 12 hours overnight fast. The adult patients were asked to ingest 2000 mL of polyethylene glycol (PEG) solution within 2 hours, starting 4 hours before swallowing the capsule. However, 30 children and adolescents were asked to ingest 1000 mL of PEG solution following the same steps. All patients who underwent CE examination provided informed written consent. Small bowel mucosal inflammation on CE was quantified using the LS by 2 independent reviewers with rich experience in small bowel CE interpretation, and they were blinded to the results of HBI, aPCDAI, and CRP. LS is an objective and validated capsule endoscopy index which was presented by Dr B.S. Lewis in 2008 primarily. It includes 3 parameters: villous oedema, ulcers, and stenosis. To calculate the LS, the small bowel transit time was divided into 3 equal parts. These parts were gauged individually. The most severe part was chosen to calculate the score of villous appearance and ulcer. Stenosis was an overall evaluation of the whole intestine. A score <135 defines as normal or clinical insignificant mucosal inflammatory change. A score between 135 (including 135) and 790 is mild. A score ≥790 is moderate–severe. SBTT defines as the time from the first duodenal image to the time of the first cercal image.

### 2.3 Clinical disease activity indices and biomarker

aPCDAI is a clinical index containing 3 history items (abdominal pain, number, and consistency of stools and patients functioning) and 3 physical examinations (weight change, abdominal mass or tenderness, and perirectal disease), which was developed from the full PCDAI in 2003 first. aPCDAI scores range from 0 to 70, with cutoff scores for remission (<10), moderate (10–25), and severe disease (≥40). HBI is a clinical index with a combination of objective and subjective items, which has been used to evaluate disease activity in adult patients with CD since 1980. It contains 5 parameters: general well-being, abdominal pain, numbers of liquid stools per day, abdominal mass, and complications. Those who scored 4 or less were considered to be in clinical remission and those with score >4 were regarded as having clinical active CD. CRP is a biological indicator which rises in the inflammatory condition. A serum CRP level below 5 mg/L was considered normal. Levels of CRP closest to the date of CE procedure were analyzed. Other than small bowel inflammation, factors which resulting in an abnormal CRP value were eliminating.

Further extraction of the following demographic and clinical characteristics were performed by checking the electronic medical records of patients: sex, birth age, age at diagnosis, CD phenotype at diagnosis according to the Montreal classification,^[22]^ disease duration, previous history of surgery and drug use, the date of CE examination and CRP analysis. These parameters are presented in Table 1.

**Table 1:**
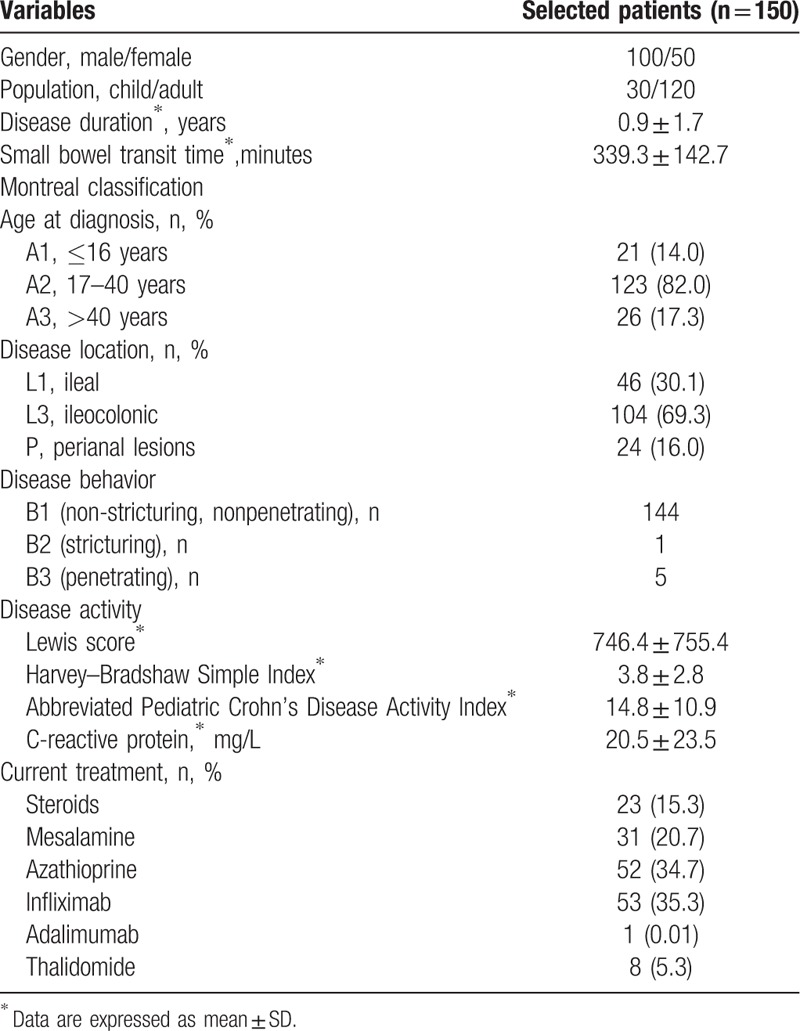
Baseline characteristics of Crohn's disease patients enrolled.

### 2.4 Data analysis

All the statistical analyses were carried out with a Statistical Package for the Social Sciences (SPSS) software version 20.0 (IBM, Armonk, NY) and Microsoft Word 2010. Results of quantitative data are expressed as mean ± SD and range. Spearman's rank correlation coefficient (*r*) was used to assess the correlation between LS and HBI and that between LS and CRP, aPCDAI, SBTT. The strength of correlation was defined as follows: *r* values ≤ 0.1 were considered to denote no correlation: 0.1 to 0.3 weak to modest; 0.3 to 0.49 moderate; 0.5 to 0.79 strong; and ≥ 0.8 very strong correlation.^[23]^ Comparisons of HBI, aPCDAI, CRP, SBTT among inactive, mild and moderate–severe CD subgroups (according to the LS) were carried out with nonparametric statistical analysis using Mann–Whitney and Kruskal–Wallis tests. Results at baseline and follow-up were compared using 2-tailed Wilcoxon analysis. A 2-tailed probability (*P*) value of less than 0.05 was considered to be statistically significant for all tests.

## 3 Results

### 3.1 Baseline characteristics

A total of 150 consecutive inpatients aged 11 to 60 years (mean 29.5 years), of whom 100 were males (66.7%), participated in the study. In the total 150 patients, 12 of 30 pediatric patients and 12 of 120 adults were found perianal lesions. In pediatric patients with small bowel CD, half of them received infliximab treatment. According to the disease distribution, 46 of them were L1 phenotype, and the rest 104 of them were L3 phenotype. All the patients completed CE examinations and CE retention was not found in them. In the study, we had not observed any adverse events such as dysphagia, nausea, and vomit. CE was well tolerated and wildly accepted in the designated patients. The LS indicated that inactive, mild, and moderate–severe patients were 4, 14, and 12 in pediatric patients and 17, 59, and 44 in adults, respectively. Table 1 shows the clinical, biological, and capsule endoscopic features of patients enrolled.

### 3.2 Correlations between LS and aPCDAI, CRP, and SBTT in pediatric patients with small bowel CD

As shown in Table 2, moderate correlations were found between LS and aPCDAI, CRP (*r*_1_ = 0.413; *r*_2_ = 0.379; *P*_1_ = .023; *P*_*2*_ = .044). There was no correlation between LS and SBTT (*r* = –0.029; *P* = .880). The correlation between CRP and aPCDAI was strong (*r* = 0.633, *P* < .001). In pediatric population, 1 patient was recorded elevated CRP level and the other 3 patients were recorded normal CRP levels in the inactive subgroup. All of them were in clinical remission. In the mild cases, 6 of 14 patients were found normal CRP levels and 3 cases were in clinical remission. In the moderate–severe patients, 11 of them were recorded normal CRP levels and all of them were found to have clinical active CD.

**Table 2:**
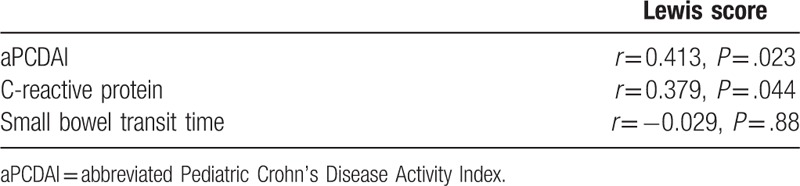
Correlations between the Lewis score and abbreviated Pediatric Crohn's Disease Activity Index, C-reactive protein, and small bowel transit time in pediatric patients with small bowel Crohn's disease.

### 3.3 Correlations between LS and HBI, CRP, and SBTT in adult patients with small bowel CD

As shown in Table 3, weak but significant correlations were found between LS and HBI, SBTT (*r*_1_ = 0.213; *r*_2_ = 0.237; *P*_1_ = .019; *P*_2_ = .009). Moderate correlation was found between LS and CRP (*r* = 0.326; *P* < .001). The correlation between CRP and HBI was strong (*r* = 0.522, *P* < .001). In adult patients, 5 of 17 patients were recorded elevated CRP levels and the rest 12 patients were recorded normal CRP levels in the inactive subgroup, and 4 patients were found to have clinical active CD. In the mild cases, 21 of 59 patients were found normal CRP levels and 36 cases were in clinical remission. In the moderate–severe patients, 24 of them were found to be in clinical remission and 13 patients were recorded normal CRP levels.

**Table 3:**
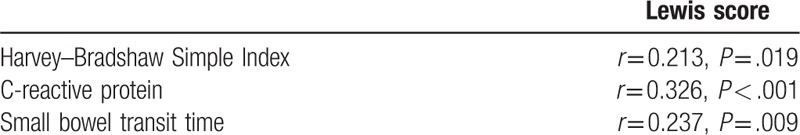
Correlations between the Lewis score and Harvey–Bradshaw Simple Index, C-reactive protein, and small bowel transit time in adult patients with small bowel Crohn's disease.

### 3.4 Comparisons of clinical disease activity indices, CRP, and SBTT among different LS subgroups

The degree of small intestinal inflammation was stratified to different subgroups by the LS. Comparisons of clinical disease activity indices, CRP and SBTT were further studied in Table 4. In pediatric patients with small bowel CD, aPCDAI and CRP could differentiate inactive subgroup from mild or moderate–severe subgroup, whereas they could not discriminate the mild subgroup from the moderate–severe subgroup (*P* > .05). In adult patients with small bowel CD, differences in HBI were not found among these subgroups. CRP levels were significantly different between the inactive and mild subgroup and that between the mild and moderate–severe subgroup. It is noteworthy that differences in SBTT among the 3 subgroups were not found both in pediatric and adult patients with small bowel CD.

**Table 4:**
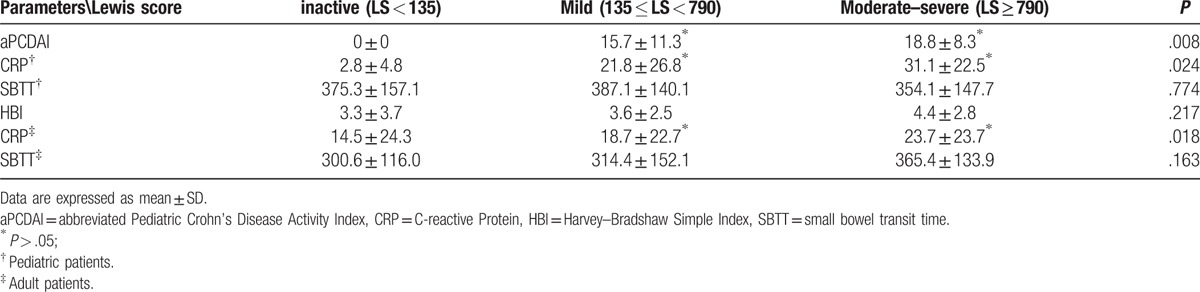
Comparisons of clinical disease activity indices, biomarker, and small bowel transit time in different subgroups.

### 3.5 Comparisons of CRP, LS, and SBTT between baseline and follow-up population who were in clinical remission after treatment

Numbers of patients who participated in the follow-up study were 10 pediatric patients and 30 adults, respectively, and they all reached clinical remission after therapy within 4 months; the average follow-up time is 3.1 months (range: 1–4 months). Comparisons of clinical disease activity indices, CRP, LS, and SBTT in adult and pediatric patients are presented in Table 5. The average clinical disease activity scores (aPCDAI and HBI) at baseline were higher than that at the follow-up (*P*_1_ = .007; *P*_2_ = .002). In pediatric patients with small bowel CD, the average LS and CRP at follow-up were lower than that at baseline (359.7 vs 727.4, 4.8 vs 21.7), whereas difference in CRP was not found to be statistically significant. There was no difference in SBTT between baseline and follow-up. In adult patients with small bowel CD, differences in LS and SBTT between baseline and follow-up were not found. Also, CRP was lower at follow-up than that at baseline with statistically significant difference (7.6 vs 22.6). Of all the follow-up patients in clinical remission, only 3 of 10 pediatric patients and 6 of 30 adults were found to achieve mucosal healing (capsule endoscopic remission, LS < 135). The average LS at follow-up were >135 in pediatric patients and >790 in adults.

**Table 5:**
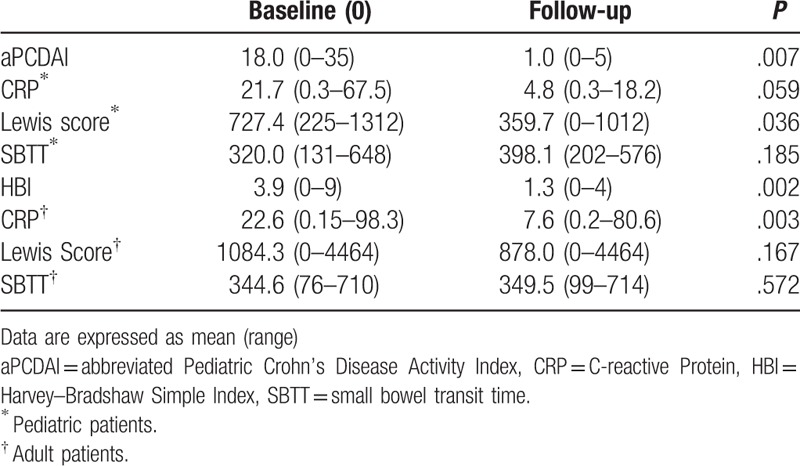
Comparisons of C-reactive protein, clinical disease activity indices, small bowel transit time, and Lewis score between baseline and follow-up.

## 4 Discussion

In our study, we analyzed the relationships between clinical disease activity indices, biomarker, and capsule endoscopic findings both in pediatric and adult patients with small bowel CD. Our results demonstrated that correlations between these parameters existed both in pediatric and adult patients, whereas the strength of these correlations were different. In all patients, strong correlation was found between clinical disease activity indices and CRP, and correlation between LS and CRP was moderate. However, correlation between clinical disease activity indices and LS was moderate in pediatric patients and weak in adults. The same study on the correlations between LS and HBI, CRP in adult patients with isolated CD was conducted by LY and his colleagues in 2013, who showed that correlation between LS and HBI was weak (*r* = 0.4, *P* < .01) and that between LS and CRP was moderate (*r* = 0.58, *P* < .01).^[24]^ However, no correlation between LS and Crohn's Disease Activity Index (CDAI), CRP in adult patients with quiescent small bowel CD was observed by Aggarwal et al^[18]^ in 2011 for the first time. Nouda et al^[20]^ analyzed 46 adult patients with CD who underwent CE and reported significantly positive correlations between the LS and CRP, CDAI (*r*_1_ = 0.431, *P*_1_ = .006; *r*_2_ = 0.508, *P*_2_ = .001) in 2013. The inconsistence of the results on correlations between clinical, biomarker, and endoscopic disease activity was presented in the previous study, and the majority of them were conducted in adults. Data on pediatric patients were relatively less. And as far as we know, the relationship between aPCDAI and LS has not yet been studied so far. In pediatric patients with CD, a positive correlation was found between CRP and PCDAI (*r*^2^ = 0.318, *P* < .0001) by Tilakaratne and his colleagues in 2010.^[25]^ However, Hoekman et al^[26]^ discovered weak correlation between CRP and aPCDAI (*r* = 0.28, *P* = .012) in 2016.

However, in the subgroups according to the LS, aPCDAI and CRP (both in pediatric and adult patients) were found to be different between inactive and mild cases and that between the inactive and the moderate–severe subgroup, with statistical significance, but difference was not found between the mild and the moderate–severe subgroup. Differences in HBI were not found among these subgroups. We suppose that clinical disease activity indices contain a lot of subjective items, which differs from individuals. The tolerance level on uncomfortableness caused by disease may be higher in adults than that in pediatric patients. Other than HBI, aPCDAI contains the item of weight loss, which is crucial in the evaluation of pediatric growth development. The cutoff scores in each of these clinical disease activity indices are different, which may exert effect on the outcome assessment. What's more, our study supports the CRP value as a biomarker of disease activity both in pediatric and adult patients with small bowel CD, it could discriminate inactive from active CD but fails to distinguish mild from moderate–severe cases, so the value of CRP level on differentiating mild from moderate–severe cases was demonstrated to be limited in our study.

Another important study was performed to analyze the relationship of SBTT with LS. Correlation between LS and SBTT was inexistence in pediatric patients and weak in adults. In the subgroups according to the LS, differences in SBTT among these groups were not found both in pediatric and adult patients. SBTT is a CE parameter, which associates with the small bowel motility. Results on the relationship between SBTT and different CD activity are conflicting. Herrerias et al^[27]^ found no difference in SBTT between patients without CE findings and those with CE suggesting CD. However, Monika Fisher and his colleges explored that prolonged SBTT was found in patients with active CD compared to patients with quiescent CD and non-IBD patients.^[28]^ According to our results, we assume that the inflammation of CD detected under CE could present as villous edema and ulcers, and the motor nerve of pathological intestinal itself may have been destroyed in the course of CD, each of these changes may exert effect on the intestinal peristalsis, but the population effect is unknown yet. Since the degrees of the small intestinal inflammation detected by CE may exhibit in various ways, which means the specific numbers and forms of the ulcers may give rise to the opposite impacts. Whether the inflammatory mediators speed up or slow down the small bowel peristalsis is not clear.

Follow-up study on the previous patients with small bowel CD was also conducted in our research. After treatment within 4 months, all this follow-up population reached clinical remission, but only the minority of them achieved mucosal healing. The average CRP level in adults and LS in children and adolescents were reduced after treatment, with statistically significant difference. However, LS at follow-up was not found to be lower than that at baseline in adult patients. The average CRP level was not improved after treatment in pediatric patients. Clinical remission may associate with the improvement of biomarker and mucosal inflammation, but it cannot determine mucosal healing. In our study, half of the pediatric patients were newly diagnosed, whereas most adult patients experienced frequent disease relapses and received prolonged treatment with various therapeutic schedules. So it seems to be more complicated to get mucosal healing in adult patients.

Limitations related to the retrospective design of the study and the heterogeneity of patients exist. The following possible drawbacks may occur in our study. Both HBI and aPCDAI contain subjective and objective parameters, and the influences of the subjective feelings and personal reaction to CRP cannot be denied. Since LS focus on the evaluation of small intestinal lesions and lacks evaluation of colonic lesions, small intestinal lesions were assessed only in the L3 phenotype of CD. So it is necessary to modify LS for gauging the whole bowel in the future.

In conclusion, attention should be paid to the capsule endoscopic evaluation both in pediatric and adult patients with small bowel CD. To reduce progressive bowel damage and monitor long-term prognosis of CD, clinical, laboratory, and endoscopic parameters should be incorporated in the management of CD. Furthermore, the small bowel transit time may not be influenced by the grade of inflammatory lesions in patients with small bowel CD. Additional reports on multicenter and prospective study are needed to confirm these preliminary results.
